# International Encyclopœdia of Surgery

**Published:** 1886-07

**Authors:** 


**International Encyclopædia of Surgery.** A systematic treatise on the theory and practice of surgery. By authors of various nations. Edited by JOHN ASHURST, JR., M. D., Professor of Clinical Surgery in the University of Pennsylvania. Illustrated with chromo-lithographs and wood cuts. In six volumes. Vol. VI. New York: Wm. Wood & Co. 1886.

The sixth and last volume of this truly great work is before us, and we congratulate its distinguished editor, not only in that his arduous labors, in this connection, are ended, but also that these labors have produced such splendid results.

We have noticed, in terms of high commendation, the successive volumes, as they have appeared, and the complete work fills us with an admiration which we believe is shared by all medical men who have the good fortune to possess the "Encyclopædia." This last volume gives a complete list of the contributors, and it is, indeed, a galaxy of famous names, and the articles in the Encyclopædia will, in almost every instance, add to the reputation of their several authors.

The articles contained in the last volume are all of superior merit. The distinguished Solis Cohen completes the consideration of the surgery of the digestive organs by a treatise on the injuries and diseases of the œsophagus. Ashurst gives an excellent article on intestinal obstructions. Allingham, of London, who deservedly ranks among the first of English proctologists, contributes an article on the injuries and diseases of the rectum. The surgery of the genito-urinary apparatus is written upon by several of the most distinguished surgeons in that department. A good deal of material follows, which was postponed from previous volumes, and the volume ends with an elaborate general index, which enables easy reference to any subject in any volume.

The publishers have done everything which the printer can do to present the work in a perfect form, and letter-press, engravings, lithographs and bindings are in every way satisfactory.

We hope that all of our readers will have the good fortune to have the Encyclopædia.

